# Mapping the Putative G Protein-coupled Receptor (GPCR) Docking Site on GPCR Kinase 2: INSIGHTS FROM INTACT CELL PHOSPHORYLATION AND RECRUITMENT ASSAYS*

**DOI:** 10.1074/jbc.M114.593178

**Published:** 2014-07-21

**Authors:** Alexandre Beautrait, Kevin R. Michalski, Thomas S. Lopez, Katelynn M. Mannix, Devin J. McDonald, Amber R. Cutter, Christopher B. Medina, Aaron M. Hebert, Charnelle J. Francis, Michel Bouvier, John J. G. Tesmer, Rachel Sterne-Marr

**Affiliations:** From the ‡ Department of Biochemistry and; the § Institute for Research in Immunology and Cancer, Université de Montréal, Montréal, Québec H3C 3J7, Canada,; the Departments of ¶ Chemistry and Biochemistry and; ‖ Biology, Siena College, Loudonville, New York 12211, and; the ** Departments of Pharmacology and Biological Chemistry, Life Sciences Institute, University of Michigan, Ann Arbor, Michigan 48109

**Keywords:** Adrenergic Receptor, Bioluminescence Resonance Energy Transfer (BRET), G Protein-coupled Receptor (GPCR), Mutagenesis, Phosphorylation, GPCR Kinase (GRK), Intact Cell Phosphorylation

## Abstract

G protein-coupled receptor kinases (GRKs) phosphorylate agonist-occupied receptors initiating the processes of desensitization and β-arrestin-dependent signaling. Interaction of GRKs with activated receptors serves to stimulate their kinase activity. The extreme N-terminal helix (αN), the kinase small lobe, and the active site tether (AST) of the AGC kinase domain have previously been implicated in mediating the allosteric activation. Expanded mutagenesis of the αN and AST allowed us to further assess the role of these two regions in kinase activation and receptor phosphorylation *in vitro* and in intact cells. We also developed a bioluminescence resonance energy transfer-based assay to monitor the recruitment of GRK2 to activated α_2A_-adrenergic receptors (α_2A_ARs) in living cells. The bioluminescence resonance energy transfer signal exhibited a biphasic response to norepinephrine concentration, suggesting that GRK2 is recruited to Gβγ and α_2A_AR with EC_50_ values of 15 nm and 8 μm, respectively. We show that mutations in αN (L4A, V7E, L8E, V11A, S12A, Y13A, and M17A) and AST (G475I, V477D, and I485A) regions impair or potentiate receptor phosphorylation and/or recruitment. We suggest that a surface of GRK2, including Leu^4^, Val^7^, Leu^8^, Val^11^, and Ser^12^, directly interacts with receptors, whereas residues such as Asp^10^, Tyr^13^, Ala^16^, Met^17^, Gly^475^, Val^477^, and Ile^485^ are more important for kinase domain closure and activation. Taken together with data on GRK1 and GRK6, our data suggest that all three GRK subfamilies make conserved interactions with G protein-coupled receptors, but there may be unique interactions that influence selectivity.

## Teaser

**Background:** Activation of GRK2 requires interaction with agonist-occupied GPCRs.

**Results:** Residues on the GRK2 N terminus and kinase domain extension collaborate to create a GPCR docking site.

**Conclusion:** Three GRK subfamilies use similar determinants to create the putative docking site, but subtle differences may dictate selectivity.

**Significance:** Mapping the GRK-GPCR interface is required to understand the mechanism and specificity of GRK activation, and, therefore, the regulation of GPCRs.

## Introduction

G protein-coupled receptors (GPCRs)^4^ are activated by a variety of extracellular signals, such as neurotransmitters, pheromones, hormones, and light, and in turn stimulate the binding of GTP to the Gα subunit of the heterotrimeric G protein (Gαβγ). Subsequently, the Gα-GTP and Gβγ components interact with downstream effectors, such as adenylyl cyclase, phospholipase C, and ion channels, to instigate intracellular signaling cascades. Upon activation, the receptors also become substrates for regulatory kinases. In vertebrates, there are seven GPCR kinases (GRKs) that can be organized into three subfamilies based on sequence similarity: GRK1 (GRK1 and GRK7), GRK2 (GRK2 and GRK3), and GRK4 (GRK4, GRK5, and GRK6). GRKs are Ser/Thr kinases that phosphorylate active GPCRs on either their third intracellular loop or, more typically, their C-terminal tail. Receptor phosphorylation promotes the binding of arrestins, which uncouple the receptor from G proteins, target the receptor for recycling or degradation, and promote ERK1/2 activation in a G protein-independent manner (1–4).

**FIGURE 1.**
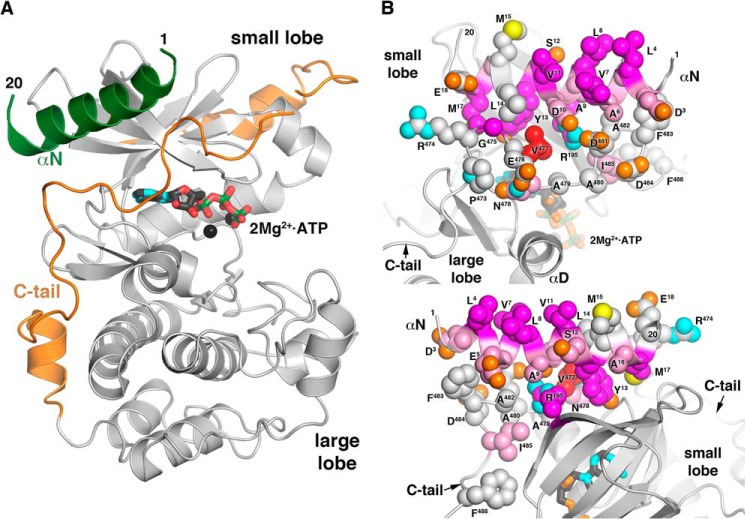
**Putative GPCR-docking site of GRK2.** *A*, homology model of the GRK2 kinase domain in an active configuration. The model was constructed primarily based on the structure of GRK6 in what is believed to be an active configuration (PDB entry 3NYN) (12). Compared with prior experimental models, the large lobe of GRK2 is rotated toward the small lobe in order to coalesce the active site found between the small and large lobes, and the N-terminal helix (αN, *green*) and the portion of the kinase domain extension (*orange*), also known as the kinase C-tail, that packs between αN and the active site (corresponding to the AST) were built using GRK6 as a guide. Only residues 1–20 at the N terminus were modeled due to ambiguities in the location of residues 21–28 and the connection to the beginning of the RH domain (not shown). Because there are as of yet no structures of GRK2 with ordered ATP in the active site, 2Mg^2+^-ATP (*stick model*) was modeled from chain B of the structure of the GRK1–2Mg^2+^-ATP complex (PDB entry 3C4W) (32). Carbon atoms are *colored dark gray*, nitrogens are *cyan*, oxygens are *red*, and phosphates are *green*. In addition to the kinase domain, GRK2 contains RH and pleckstrin homology domains (not shown). *B* and *C*, summary of the functional analysis of the proposed receptor-docking site. Residues modeled to pack between αN and the small lobe are the most critical for phosphorylation of Rho *in vitro* of β_2_AR in intact cells and for recruitment to α_2A_AR as measured by BRET. The side chains of residues that were targeted by site-directed mutagenesis in this and a prior study (22) are shown as *spheres* whose carbons are *colored* using an intensity scale corresponding to the degree of loss or gain of function in BRET recruitment (Asp^3^, Leu^4^, Val^7^, Leu^8^, Asp^10^, Val^11^, Ser^12^, Tyr^13^, Met^15^, Ala^16^, Met^17^, Glu^18^, Arg^195^, Gly^475^, Val^477^, Asp^481^, Ala^482^, and Ile^485^) and Rho phosphorylation (Glu^5^, Ala^6^, Ala^9^, Leu^14^, Arg^474^, Gly^475^, Glu^476^, Asn^478^, Ala^479^, Ala^480^, Phe^483^, Asp^484^, and Phe^488^) assays (*red* (biggest effect) > *magenta* > *pink* > *gray* (no significant difference). The side chains of Tyr^13^ from αN and Val^477^ from the kinase extension pack together and against the small lobe, forming a small hydrophobic core for the docking site. Hydrophobic residues Leu^4^, Val^7^, Leu^8^, Val^11^, and polar Ser^12^ form another significant “hot spot” along the top of the modeled αN helix, which is proposed to interact with activated GPCRs (see Ref. 13 and this work). The view in *C* is rotated from that of *B* by ∼180° around a roughly vertical axis. Oxygen atoms are *colored orange*, nitrogens are *cyan*, and phosphates are *green*.

GRKs belong to the AGC kinase family (kinase domain related to protein kinases A, G, and C), and thus their kinase domains have many characteristics in common with the catalytic domain of protein kinases A, B (Akt), and C (5). However, GRKs have evolved unique features required for recognition of and activation by their GPCR substrates. In addition to the kinase domain, GRKs have an RGS (regulator of G protein signaling) homology (RH) domain, which is thought to serve as a scaffold that helps stabilize an active configuration of the small lobe of the kinase domain (6–8). In addition, all GRKs contain an N-terminal ∼20-residue segment that is predicted to have helical character (αN) and is essential for receptor phosphorylation (9–13) (Fig. 1*A*).

Membrane localization is also key to GRK function, and the various GRKs utilize individual mechanisms to promote membrane localization (4). For example, GRK2/3 have a C-terminal pleckstrin homology domain that binds both Gβγ and anionic phospholipids and that is absolutely required for the agonist-dependent recruitment of GRK2 to the site of an activated GPCR (14–16) and for receptor desensitization (17).

Receptor binding and GRK activation are expected to be intrinsically coupled events (18). At least three GRK structural regions are proposed to be involved in this activation mechanism: the N-terminal region (9, 13), the active site tether (AST) in the kinase domain extension of the AGC kinase domain (19–22), and surface residues on the small lobe of the kinase domain (21) (Fig. 1, *B* and *C*). Most GRK crystal structures reported thus far display unstructured N-terminal and kinase domain extension regions, and their kinase domains adopt inactive conformations. The sole exception is the GRK6-sangivamycin complex, wherein the small and large lobes assume a conformation similar to that of activated PKA, and the N-terminal region forms an α-helix (αN) that packs against the small lobe and the AST (12). This structure and a cross-linking study (13) suggest that activated GPCRs could activate GRKs by ordering the αN and AST regions, which in turn stabilizes the small and large lobes in their active configuration. However, the sequence of these events is not clear.

Prior studies of GRK1, GRK2, and GRK6 demonstrated that some mutations in the N-terminal region exhibit defects in receptor but not in non-receptor substrate phosphorylation (10, 11), whereas mutations in the small lobe and AST involved in packing with the αN exhibit severe defects in the phosphorylation of both types of substrates (12, 21). These results are consistent with the idea that the N-terminal helix may interact directly with activated receptors, whereas the AST is more important for coupling receptor binding to kinase domain closure (13, 22). However, the AST region of GRK2 exhibits greater sequence divergence than the rest of the kinase domain does from GRK6; therefore, residues in the GRK2 N-terminal and AST regions may play different roles in stabilizing the closed state of the kinase domain or be of differential importance for interacting with GPCRs. Although Wang *et al.* (23) and Baameur *et al.* (8) have identified RH domain residues that may play a role in receptor and non-receptor phosphorylation, the focus of this study is on the αN and AST regions. We mutagenized residues 3–18 of GRK2 and additional residues of its AST. The catalytic capabilities of these variants were assessed *in vitro* and in intact cells. To directly test the role of GRK kinase domain extension and N-terminal mutants in recruitment to activated GPCRs, we developed a bioluminescence resonance energy transfer (BRET) assay (24, 25) that measures the norepinephrine (NOR)-induced recruitment of GRK2 to the vicinity of the α_2A_-adrenergic receptor (α_2A_AR). Our results suggest that, as predicted for GRK1 and GRK6 (12, 21), the N-terminal and kinase domain extension residues of GRK2 collaborate with the kinase small lobe to form the allosteric docking site on GPCRs. However, our results indicate that the relative importance of individual residues differs. These differences probably contribute to receptor selectivity.

## EXPERIMENTAL PROCEDURES

### Materials

African green monkey kidney cells (COS-7) were from the American Tissue Culture Collection (ATCC). N-terminal FLAG-tagged human β_2_AR cDNA in a mammalian cell expression vector (pcDNA3-FLAG-β_2_AR) was a gift from Dr. Jeffrey Benovic (Thomas Jefferson University, Philadelphia, PA) (26). pcDNA3.1-HA-β_2_AR(Y326A) was a gift from Drs. Marc Caron and Larry Barak (27, 28) (Duke University, Durham, NC). pcDNA3.1-Gβ and pcDNA3.1-Gγ were purchased from the Missouri University Science and Technology cDNA Resource Center, and bovine GRK2 cDNA in a mammalian cell expression vector, pcDNA3-GRK2 wild type (WT) and K220R (29), were provided by Dr. Jeffrey Benovic. [γ-^32^P]ATP was from MP Biomedical or PerkinElmer Life Sciences, FuGENE-HD was from Roche Applied Science or Promega, isoproterenol was from Sigma, and peptide *N*-glycosidase F was from New England Biolabs. Polyclonal antibodies recognizing β_2_AR Ser(P)^355^/Ser(P)^356^, the β_2_AR carboxyl tail (to detect total receptor), and GRK2 were obtained from Santa Cruz Biotechnology, Inc. Protein structure figures were generated using the PyMOL Molecular Graphics System.

### Homology Model of Activated GRK2

The structure of GRK6 in complex with sangivamycin (PDB entry 3NYN) (12) was used as a template. First, the small lobe of bovine GRK2 (PDB entry 1OMW) (6) was aligned with the small lobe of GRK6, and then residues 277–470 of the GRK2 large lobe were superimposed on the analogous residues of GRK6. GRK2 residues 3–18 (N-terminal helix) and residues 471–489 (AST) were built by hand using the corresponding regions of GRK6 as a template for the backbone. GRK2 residues 1–2 and 19–20 were then built as N- and C-terminal extensions to the N-terminal helix. GRK2 residues 492–497 were modeled based on PDB entries 3NYN and 3KRW (30). The linker between the large and small lobe and the hand-built regions were then minimized to idealize stereochemistry. All modeling was performed using the program O (31).

### Expression Construct Design and Mutagenesis

Variants of pFastBac-Dual-GRK2-H_6_ (pFBD-GRK2-H_6_) (21, 32, 33) were generated using QuikChange (Stratagene/Agilent) with slight modifications. Mutations were generated in the N-terminal region (D3A, L4A, E5A, A6N, V7A, V7E, L8A, A9V, D10A, D10R, V11A, S12A, Y13A, L14A, M15A, A16V, M17A, and E18A) and kinase domain AST (R474A, G475I, V477D, N478A, A479S, A480S, A482I, and I485A). pcDNA3-GRK2 variants K220R, V477D, and I485A were described previously (22, 29), but variants D3A, L4A, V7E, D10R, Y13A, M15A, A16V, and M17A were generated for this work. The RlucII and GRK2 coding sequences for the RlucII-GRK2 construct were PCR-amplified from RlucII-β-arrestin1-GFP10 (34) and human GRK2 cDNA (OpenBiosystems, clone ID 6203199), respectively. The two fragments were then fused by overlapping PCR and inserted between NheI/BsiWI in the pIREShyg3 vector (Clontech). The amino acid sequence of the linker between the RlucII and GRK2 is GGSGSGSGS. For RlucII-GRK2 mutants (D3A, L4A, V7A, V7E, V7P, L8E, V7E/L8E, D10A, D10R, V11A, S12A, Y13A, M15A, A16V, M17A, E18A, R195A, K220R, D317A, P473E, G475I, V477D, D481A, A482I, I485A, and R587Q), QuikChange or PCR-based mutagenesis was used to substitute specific codons in the GRK2 open reading frame. The generation and functionality of α_2A_AR-GFP^2^ were described previously (35). All constructs were confirmed by DNA sequencing.

### Expression of GRK2-H_6_ in High 5 Insect Cells

The Bac-to-Bac Expression System (Invitrogen) was used to generate baculoviruses from the various pFBD-GRK2-H_6_ constructs. For GRK2-H_6_ expression, High 5 cells (9 × 10^6^) were plated on 10-cm tissue culture plates in SF900 II medium containing 100 units/ml penicillin, 100 μg/ml streptomycin, and amphotericin B and infected for 48 h with appropriate GRK2 variant baculovirus (33). Cells were collected at 500 × *g*, washed three times in STE (20 mm Tris, 150 mm NaCl, 1 mm EDTA, pH 8) before suspension in 0.4 ml lysis buffer (20 mm HEPES, pH 8, 250 mm NaCl, 0.02% Triton X-100, 0.5 mm phenylmethylsulfonyl fluoride (PMSF), 0.2 mg/ml benzamidine, 10 μg/ml leupeptin, 1 mm dithiothreitol (DTT). Cells were lysed by micro-tip sonication using 90 cycles of alternating 1-s bursts and 2-s rest periods, and then lysates were clarified by centrifugation for 20 min at 40,000 × *g*. High-speed supernatants were stored in 25% glycerol at −20 °C. GRK2 content in each lysate was determined by Western blot comparison with a GRK2 standard curve.

### Rhodopsin Phosphorylation by High 5 Lysates Containing GRK2-H_6_ Mutants

High 5 cell lysates with overexpressed GRK2 were screened in a rhodopsin (Rho) phosphorylation assay to identify mutants defective in GPCR phosphorylation. Rod outer segments were isolated from frozen dark-adapted bovine retinas (W. L. Lawson Co., Lincoln, NE), washed with urea as described previously (36), and resuspended in kinase assay buffer (20 mm Tris-HCl, 2 mm EDTA, 7.5 mm MgSO_4_, pH 7.5). Reactions (12.5 μl) were performed in kinase assay buffer containing 8 μm Rho, 200 μm [γ-^32^P]ATP (∼1 dpm/fmol ATP), and 2 μl of each lysate. Reactions were equilibrated at 30 °C for 30 s under dim red light illumination before incubation in ambient fluorescent light. Reactions were quenched after 3 min with 12.5 μl of SDS-PAGE sample buffer (containing 50 mm DTT), and then samples were incubated for 30 min at 65 °C with periodic vortex mixing before separating Rho by 10% SDS-PAGE. Gels were stained with Coomassie Blue, and ^32^P incorporation into Rho bands was determined by liquid scintillation counting. The activity of a High 5 lysate prepared from uninfected cells was subtracted from all samples to account for the activities of endogenous kinases (typically ∼2% of WT GRK2).

### Purification of Recombinant GRK2

GRK2-H_6_ variants in High 5 lysates that displayed reduced catalytic activity toward Rho were purified to homogeneity, as described in detail (33), for further analysis. Cultures of log phase High 5 cells (∼250 ml of ∼100 × 10^4^ cells/ml) in antibiotic/antimycotic-containing Sf900 II medium were infected with GRK2 baculovirus (volume previously optimized) and incubated at 28 °C for 48–60 h. Cells were collected; washed in STE; resuspended in Ni^2+^-column buffer (NCB) (20 mm HEPES, pH 8, 300 mm NaCl, 1 mm DTT, 1 mm PMSF, 10 μg/ml leupeptin, and 0.2 mg/ml benzamidine); lysed with a Polytron tissue disruptor; and centrifuged at 35,000 × *g* for 20 min at 4 °C. Supernatants were centrifuged again for 45 min at 100,000 × *g*, and the resulting supernatants were diluted to 5 mg/ml protein with NCB and adjusted to 20 mm imidazole before loading onto a 5-ml column (Bio-Rad, equipped with a flow adaptor) containing Ni^2+^-nitrilotriacetic acid-agarose (Qiagen). Following 15-ml washes with NCB plus 20 mm imidazole and NCB plus 40 mm imidazole, the column was eluted with 40 ml of NCB plus 150 mm imidazole. Fractions (1 ml) were collected and analyzed by Coomassie staining of 8% SDS-polyacrylamide gels. GRK2-H_6_-containing fractions were pooled, diluted to a NaCl concentration of 50 mm with High S column buffer (HCB; 20 mm HEPES, 5 mm EDTA, pH 8, 0.02% Triton X-100, 50 mm NaCl, 1 mm DTT, 1 mm PMSF, 10 μg/ml leupeptin, and 0.2 mg/ml benzamidine), and loaded onto tandem 1-ml High Q/High S columns (Bio-Rad). The High Q column was removed, and the washed High S column was eluted with a 20-ml linear gradient of 50–600 mm NaCl in HCB. Triton X-100 was omitted from HCB for the purification of AST mutants, R474A, G475I, N478A, A479S, and A480S. Fractions (0.5 ml) were collected, analyzed by Coomassie staining of 8% SDS-polyacrylamide gels, pooled, and, when necessary, concentrated by centrifugation at 2500 × *g* using Centricon (Amicon) filtration. Purified proteins were stored in 25% glycerol at −80 °C, and GRK2 content was determined by Coomassie staining of 8% SDS-polyacrylamide gels.

### Kinase Assays with Purified GRK2-H_6_ Variants

#### 

##### Rho Phosphorylation

Phosphorylation of Rho was performed in 10-μl reactions containing 20 nm GRK2, 8 μm Rho, 200 μm [γ-^32^P]ATP (∼1 dpm/fmol) in GRK2 kinase buffer. Reactions were terminated with 14 μl of SDS-PAGE sample buffer containing 50 mm DTT and processed as described above. The kinase activity observed in the absence of GRK2 (uninfected lysates or a buffer control) represented 2–7% of WT-GRK2 activity and was subtracted from all samples.

##### Peptide C Phosphorylation

The phosphorylation of peptide C (DDEASTTVSKTETSQVARRR), corresponding to the carboxyl tail of bovine rhodopsin, was measured in 10μl reactions containing 200 nm GRK2 variant, 100 μm [γ-^32^P]ATP (∼1.5 dpm/fmol), and 1 mm peptide C in kinase assay buffer. Reactions were incubated for 50 min at 30 °C and quenched with 14 μl of SDS-PAGE sample buffer containing 10 mm DTT. Samples were resolved by 18% SDS-PAGE following incubation at 65 °C for 10 min. Peptide C bands were excised and measured for ^32^P incorporation by scintillation counting.

##### Receptor-mediated GRK Activation Assay

Receptor-dependent activation of peptide C phosphorylation by GRK2-H_6_ variants was measured using endoproteinase Asp-N-treated Rho (^329^G-Rho), which lacks its carboxyl tail phosphorylation sites (18). Reactions (12 μl) containing ∼100 nm GRK2, 100 μm [γ-^32^P]ATP (∼1.5 dpm/fmol), ∼2 μm ^329^G-Rho, and 100 μm peptide C in GRK2 kinase assay buffer were equilibrated at 30 °C for 5 min before exposure to ambient light or continued incubation in the dark for 30 min. SDS-PAGE sample buffer (12 μl) containing 10 mm DTT was used to quench each reaction. Samples were incubated for 10 min at 65 °C and resolved by 18% SDS-PAGE, and the excised peptide C bands were used to measure ^32^P incorporation as described above. Relative to GRK2 purified in the presence of 0.02% Triton X-100, WT GRK2 purified in its absence displayed a 11-fold decrease in the ability to phosphorylate Rho. Therefore, GRK2 activity presented in Fig. 4 for WT and GRK2 variants R474A, G475I, N478A, A479S, and A480S was normalized accordingly.

### Michaelis-Menten Kinetics

*K_m_* and *k*_cat_ were determined by varying Rho concentration in rod outer segments between 2.5 and 30 μm in 10-μl reactions containing 100 nm GRK2-H_6_ variant, 100 mm HEPES, pH 7.5, 1 mm EDTA, 10 mm MgCl_2_, and 1 mm [γ-^32^P]ATP (∼0.2 dpm/fmol). Phosphorylated Rho was quantified as described above, and initial velocities were fit to the Michaelis-Menten rate equation using GraphPad Prism version 4.0.

### β_2_AR Phosphorylation by WT and GRK2 Mutants in Intact Cells

GRK2-dependent phosphorylation of β_2_AR in COS-7 cells was carried out as described (33). Briefly, COS-7 cells were grown in DMEM (Lonza, Invitrogen) supplemented with fetal bovine serum (10%), penicillin/streptomycin/amphotericin B (Fungizone; 100 units/ml), and l-glutamine (4 mm) at 37 °C with 5% CO_2_. Cells (3.0 × 10^5^ cells/well) were plated in 6-well tissue culture dishes and transfected the following day with 0.8 μg of pcDNA3.1-FLAG-β_2_AR(WT) or pcDNA3.1-HA-β_2_AR(Y326A) and 0.4 μg each of pcDNA3.1-Gβ, pcDNA3.1-Gγ, and pcDNA3-GRK2 using 8 μl of FuGENE-HD. After 48 h, cells were serum-starved for 30 min before a 5-min treatment with 10 μm isoproterenol (ISO) or alprenolol, as indicated. Cells were washed twice with cold 20 mm Tris, pH 7.5, 150 mm NaCl and scraped with 200 μl/well receptor solubilization buffer (20 mm HEPES, pH 7.4, 150 mm NaCl, 10 mg/ml dodecylmaltoside, 10 mm DTT, 1 mm PMSF, 10 μg/ml leupeptin, 200 μg/ml benzamidine, 20 mm tetrasodium pyrophosphate, and 10 mm NaF). The resulting lysates were sonicated using a 30-cycle regimen of 1 s on/2 s off, and the receptor was further solubilized by mixing for 30 min on an orbital shaker at 4 °C. We have subsequently determined that sonication is unnecessary. Lysates were clarified by centrifugation at 16,000 × *g* at 4 °C, and 30 μl of the soluble fractions were treated with 100 units of peptide *N*-glycosidase F for 2 h at 37 °C. Peptide *N*-glycosidase F-treated samples were resolved by 10% SDS-PAGE and transferred to nitrocellulose, and immunoblotting was performed sequentially with three primary antibodies: 1) β_2_AR phosphosite antibody that recognizes agonist-induced phosphorylation (Ser(P)^355^/Ser(P)^356^); 2) an antibody that recognizes the β_2_AR carboxyl tail and reflects total β_2_AR; and 3) GRK2 polyclonal antibody. Each primary antibody was visualized with horseradish peroxidase-conjugated goat anti-rabbit secondary antibody and chemiluminescent substrates, either SuperSignal West Femto for Ser(P) blots or SuperSignal West Pico for β_2_AR and GRK2 immunoblots. Signals were visualized using the Bio-Rad ChemiDoc XRS System, and band intensities were quantified using Bio-Rad Quantity One software. Stripping of antibodies from nitrocellulose between immunoblotting experiments was achieved by incubation in 25 mm glycine pH 2, 1% SDS for 30 min at room temperature.

### BRET-based Assay of GRK2 Recruitment to α_2A_AR

HEK293T cells were cultured in DMEM supplemented with 10% FBS, 100 units/ml penicillin and streptomycin (Wisent Inc.) and incubated at 37 °C in 5% CO_2_. Two days before the experiments, the polyethyleneimine 25-kDa linear transfecting agent (PEI; Polysciences) was used with a 3:1 PEI/DNA ratio to transfect either 2.5 μg of total DNA into 10^6^ cells in 6-well plates (for one-point stimulation and titration experiments) or 12 μg of total DNA into 6 × 10^6^ cells in 10-cm dishes (for kinetics and dose-response experiments). α_2A_AR-GFP^2^ and RlucII-GRK2 DNA constructs were diluted in 150 mm NaCl in Tris-HCl (150 mm, pH 7.4), and the total amount of DNA transfected in each well or dish was equalized with salmon sperm DNA (Invitrogen). On the day of the BRET experiment (48 h post-transfection), cells were washed, detached, resuspended in PBS, and distributed (10^5^ cells/well) to 96-well microplates (White Optiplate; PerkinElmer Life Sciences). The expression level of α_2A_AR-GFP^2^ was measured as total fluorescence using a FlexStationII (Molecular Devices) with excitation and emission filters at 395 and 510 nm, respectively. Cells were then incubated in PBS or NOR at room temperature. For one-point stimulation and titration experiments, cells were stimulated with 100 μm NOR for 6 min. For time courses, 100 μm NOR was dispensed for the indicated times. Dose-response curves were performed 6 min after NOR treatment at the indicated concentrations. For all BRET experiments, 2 min before BRET readings, coelenterazine 400A (Biotium) was added to a final concentration of 5 μm, and the expression level of the RlucII-GRK2 variants was measured using a Mithras LB940 multidetector plate reader (Berthold Technologies). BRET measurements were collected using the same plate reader by recording signals detected in the 480 ± 20- and 530 ± 20-nm windows for the *Renilla* luciferase and GFP^2^ light emissions, respectively. To determine the specific BRET signal (net BRET), the background signal detected in cells transfected with the luciferase donor alone was subtracted from the BRET values obtained in cells expressing both the energy donor and acceptor. Agonist-promoted BRET reflects the difference between the 530/480 ratios in the presence and absence of NOR. Except for titration experiments, BRET experiments were performed at equivalent acceptor/donor expression ratios for all GRK2 constructs tested. For BRET titrations, a constant amount of the donor was cotransfected with increasing amounts of the acceptor, and for each transfection condition, BRET values were expressed as a function of the total expression level of the acceptor over the total expression level of the donor detected.

### Statistical Analysis

Statistical significance of differences between WT and mutants in phosphorylation assays was assessed with GraphPad Prism version 4.0 software using one-way or two-way analysis of variance (ANOVA) followed by *a posteriori* Dunnett's test. For BRET assays, statistical significance was evaluated based on one-way ANOVA with a Tukey's post-test following pooling of all the data.

## RESULTS

### 

#### 

##### Survey of GRK2 N-terminal Mutations for Their Ability to Phosphorylate Rhodopsin

**FIGURE 2.**
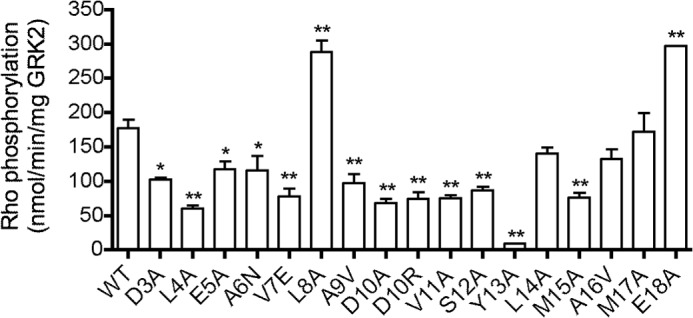
**Survey of GRK2 N-terminal variants for their ability to phosphorylate rhodopsin.** Soluble fractions from baculovirus-infected High 5 cell lysates were used in an initial screen for kinase activity. Reactions were performed at 30 °C for 3 min with [γ-^32^P]ATP (200 μm) and Rho (8 μm) as substrates and stopped with SDS sample buffer. Rho was resolved by SDS-PAGE, and Coomassie-stained bands were excised and subjected to scintillation counting. The mean ± S.E. (*error bars*) from 3–12 independent experiments are presented. One-way ANOVA determined statistical significance compared with WT (*, *p* < 0.05; **, *p* < 0.01).

**FIGURE 3.**
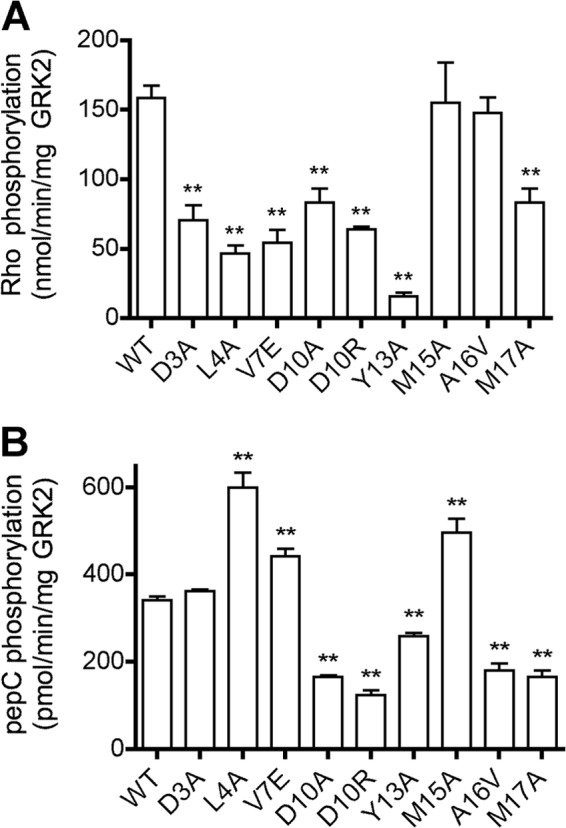
**Characterization of GRK2 N-terminal variants by *in vitro* kinase assays.** *A*, rhodopsin phosphorylation. Purified WT and GRK2-H_6_ variants (20 nm) were assessed for their ability to phosphorylate Rho as described in Fig. 2. The mean ± S.E. (*error bars*) from 3–14 independent experiments for each variant is presented. *B*, peptide C phosphorylation. Purified WT and GRK2-H_6_ variants (200 nm) were incubated for 50 min with peptide C (1 mm) and [γ-^32^P]ATP (100 μm). The values represent the mean ± S.E. from five independent experiments. One-way ANOVA was used to compare statistical significance *versus* WT. **, *p* < 0.01.

The GRK6-sangivamycin crystal structure showed that the extreme N terminus of the enzyme can fold as an extended helix that packs in a cleft formed between the kinase small lobe and the AST. Because point mutations in these interfaces impair function in GRK1, GRK2, GRK5, and GRK6 (10, 12, 13, 21, 22), it was proposed that this interaction is necessary to form the receptor-docking site on GRKs (37). Previous studies of GRK2 have not systematically tested residues in the proposed receptor-docking site. Thus, we mutated nearly all of the residues in the GRK2 N terminus (positions 3–18) and generated additional mutants in its AST to better assess the extent and the role of specific GRK2 residues in forming the receptor-docking site. As an initial survey to identify residues for detailed characterization, GRK2-H_6_ variants were expressed in High 5 insect cells, and cell lysates were screened for their ability to phosphorylate light-activated Rho (Fig. 2). The Y13A mutation resulted in a loss of 95% of activity relative to WT. Other mutants, L4A, V7E, A9V, D10A, D10R, V11A, S12A, and M15A, lost 50–65% activity. Interestingly, two mutants, L8A and E18A, seemed to potentiate Rho phosphorylation by ∼65%. A subset of the GRK2 mutants (D3A, L4A, V7E, D10A, D10R, Y13A, M15A, A16V, and M17A) was selected for further analysis and purified to homogeneity, and the Rho phosphorylation was re-evaluated (Fig. 3*A*). Most of the purified mutants showed defects comparable with those of their counterparts in cell lysates with the exception of the M15A mutant, which did not exhibit a significant defect when purified. Conversely, M17A showed a more pronounced defect when purified. The reason for this discrepancy is not clear. Of the purified N-terminal mutants, the Y13A mutation continued to show significantly diminished activity compared with WT (90% reduced). Most other mutants lost 50–70% activity (Fig. 3*A*).

##### Receptor-independent Phosphorylation of Peptide C

Phosphorylation of peptide substrates is thought to measure the intrinsic ability of the GRK kinase domain to adopt its active closed conformation, independent of receptor binding. Therefore, purified GRK2-H_6_ variants were assayed for their ability to phosphorylate peptide C, a synthetic substrate derived from the cytoplasmic tail of Rho (Fig. 3*B*). The D10A, D10R, Y13A, A16V, and M17A mutants lost 47–76% of WT activity. Because these positions are predicted to be oriented at the interface with the small lobe (Ala^16^ and Met^17^) or with the AST (Asp^10^ and Tyr^13^) (Fig. 1*B*), their reduced activity is consistent with the N-terminal helix stabilizing the active state of the GRK2 kinase domain. In contrast, the L4A and V7E variants, which were significantly impaired in Rho phosphorylation, retained 176 and 130% of WT activity, respectively. In the homology model, the Leu^4^ and Val^7^ side chains point away from the small lobe and AST and are positioned to make intermolecular interactions (Fig. 1*B*). Our results are consistent with the hypothesis that these GRK residues make direct interactions with GPCRs (12, 13).

##### Michaelis-Menten Kinetics

To better understand the roles of the WT residues in GRK2 function, Michaelis-Menten kinetic constants were measured for selected GRK2-H_6_ N-terminal variants (L4A, D10R, and Y13A) using light-activated Rho as the substrate. The L4A mutation resulted in a 5-fold *K_m_* defect with retention of *k*_cat_ (Table 1). A *K_m_* but not a *k*_cat_ defect is consistent with Leu^4^ directly interacting with receptors. Conversely, the D10R variant exhibited a 2.8-fold defect in *k*_cat_ and a 1.9-fold defect in *K_m_*, suggesting that Asp^10^ is involved in the allosteric mechanism of GRK2 activation, perhaps via its direct contacts with the AST. Y13A exhibited a 30-fold decrease in catalytic efficiency, *k*_cat_*/K_m_*, due to 6- and 5-fold defects in *k*_cat_ and *K_m_*, respectively, consistent with the fact that Tyr^13^ is predicted to pack between the small lobe and the N-terminal helix (Fig. 1*B*).

**TABLE 1:** **Michaelis-Menten kinetic parameters for GRK2 N-terminal mutants** Phosphorylation reactions were carried out with Rho (2.5–30 μm), 100 nm GRK2-H_6_ variant, [γ-^32^P]ATP (1 mm, 0.2 dpm/fmol) in 100 mm HEPES, pH 7.5, 10 mm MgCl_2_, 1 mm EDTA for 2 min under white light. Initial velocities from six experiments were fitted to the Michaelis-Menten rate equation to derive *k*_cat_ and *K_m_* values ± S.E.

GRK2 variant	*k*_cat_	*k*_cat_ decrease	*K_m_*	*K_m_* increase	*k*_cat_/*K_m_*	*k*_cat_/*K_m_* decrease
	*s*^−*1*^	*-fold*	*m* × *10*^−*6*^	*-fold*	*m*^−1^ *s*^−1^ × *10^4^*	*-fold*
WT	1.01 ± 0.11	1.0	10.7 ± 2.8	1.0	9.5	1
L4A	0.91 ± 0.32	1.1	51.1 ± 25	4.8	1.8	5.4
D10R	0.36 ± 0.07	2.8	20.2 ± 7.0	1.9	1.8	5.3
Y13A	0.18 ± 0.05	5.7	54.8 ± 20	5.1	0.32	29

##### Characterization of AST Mutants

**FIGURE 4.**
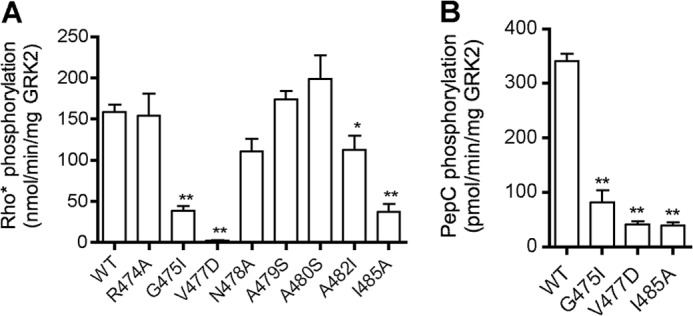
**Characterization of GRK2 AST variants by *in vitro* kinase assays.** *A*, Rho phosphorylation (mean ± S.E. (*error bars*) from 5–14 independent experiments). *B*, peptide C phosphorylation (mean ± S.E. from 3–10 independent experiments). Reactions were carried out as described in the legend to Fig. 3. One-way ANOVA was used to compare statistical significance *versus* WT. *, *p* < 0.05; **, *p* < 0.01.

The GRK2 AST region extends from residue 475 to 485. The G475I, V477D, and I485A mutants displayed a 75–98% reduction in Rho phosphorylation (Fig. 4*A*) and 76–88% reduction in peptide C phosphorylation (Fig. 4*B*). Thus, these positions are important for both receptor and peptide phosphorylation, consistent with the interactions these residues are predicted to form with the small lobe and/or N-terminal helix. Notably, variants N478A, A479S, A480S, and A482I, whose side chains point toward the kinase large lobe, were not impaired in Rho phosphorylation (Fig. 1*B*).

##### Receptor-stimulated Phosphorylation of Peptide C by GRK2 Variants

**FIGURE 5.**
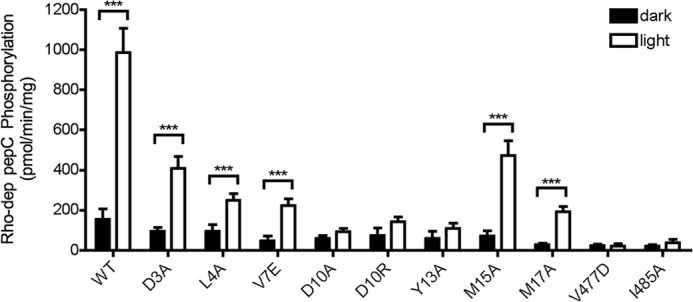
**Receptor-mediated GRK2 activation.** The ability of ^329^G-Rho (2 μm) to promote GRK2 (100 nm) activation was determined by measuring phosphorylation of peptide C (100 μm) in the presence of 100 μm [γ-^32^P]ATP with or without light for 30 min. Peptide C phosphorylation was quantified as described in the legend to Fig. 3. The mean ± S.E. (*error bars*) from 3–4 independent experiments are shown. Two-way ANOVA was used to compare statistical significance of dark *versus* light samples. ***, *p* < 0.001.

We next measured the ability of C-terminally truncated, light-activated Rho (Rho*) to accelerate phosphorylation of peptide C by our GRK2 variants. Whereas ^329^G-Rho* stimulated WT activity 6.4-fold, it stimulated D10A, D10R, and Y13A less than 2-fold and did not activate the V477D and I485A mutants (Fig. 5). The N-terminal mutants whose side chains are positioned to interact directly with receptor, D3A, L4A, and V7E, were activated only 2–4-fold by ^329^G-Rho*. Overall, these results indicate that all of the tested positions contribute to allosteric activation of peptide phosphorylation by receptors. The fact that the latter set of variants were not deficient in peptide C phosphorylation suggests once again that these αN residues are involved in direct interactions with receptor.

##### Phosphorylation of β_2_AR(Y326A) by GRK2 Variants in Intact Cells

**FIGURE 6.**
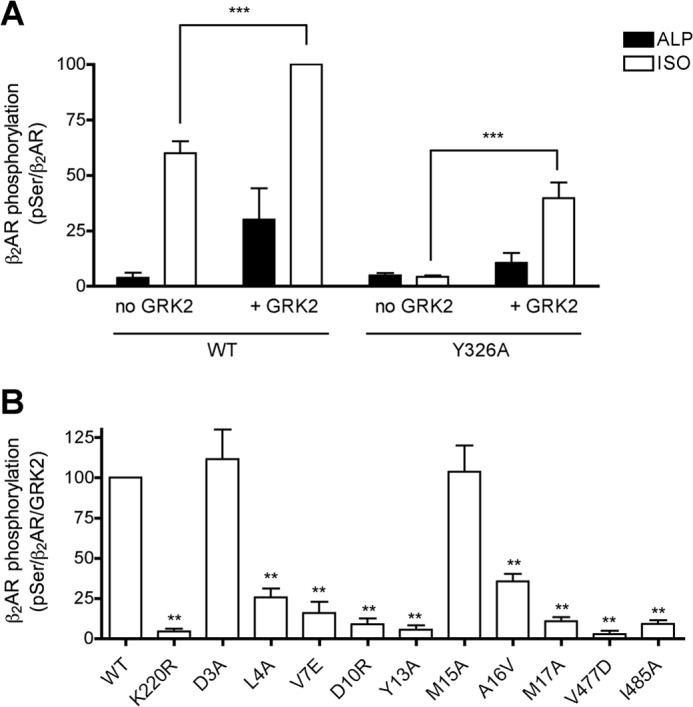
**GRK2-dependent phosphorylation of β_2_AR by GRK2 variants in COS-7 cells.** *A*, β_2_AR (WT or Y326A variant), Gβ, and Gγ were co-transfected in the presence or absence of GRK2 (WT), treated with agonist ISO or antagonist alprenolol (*ALP*), and analyzed by immunoblotting. Phosphorylated β_2_AR was detected with a GRK phosphosite antibody (*pSer*), and total β_2_AR was detected with an antibody that recognizes the carboxyl tail of the receptor (β*_2_AR*). The level of phosphorylated β_2_AR was normalized to the level of total receptor. Two-way ANOVA was used to compare statistical significance of differences between samples transfected without and with GRK2. ***, *p* < 0.001. *B*, β_2_AR(Y326A), Gβ, and Gγ were co-transfected with WT or GRK2 variants, treated with isoproterenol, and analyzed by immunoblotting as described in *A.* The level of phosphorylation was normalized to the levels of both total receptor and GRK2 and expressed as a percentage of WT. Mean ± S.E. (*error bars*) from four independent experiments are shown. One-way ANOVA was used to compare statistical significance *versus* WT. **, *p* < 0.01.

To determine whether defects observed *in vitro* occur in intact cells, we first assessed the agonist and GRK2 dependence of β_2_AR phosphorylation in COS-7 cells co-expressing β_2_AR/GRK2/Gβγ using a GRK phosphosite immunoblotting assay (33, 38) (Fig. 6*A*). We previously demonstrated that Gβγ enhanced the detection of GRK2-dependent phosphorylation of β_2_AR (33), consistent with the absolute requirement of GRK2-Gβγ complex formation for receptor phosphorylation (17). In the absence of transfected GRK2, 10 μm ISO stimulated β_2_AR phosphorylation 16-fold over the level detected in alprenolol-treated cells. However, when additionally co-transfected with GRK2, phosphorylation of β_2_AR in the absence of ISO was stimulated 8-fold, and the ISO-induced phosphorylation only increased by 40%. Thus, the high background contributed by endogenous kinases precludes the use of WT β_2_AR as a substrate to distinguish the role of GRK2 variants in their ability to promote receptor phosphorylation. It was previously demonstrated that the β_2_AR(Y326A) variant is totally impaired in ISO-dependent phosphorylation by endogenous GRKs but that overexpression of GRK2, GRK3, or GRK5 can rescue this defect (27, 39). Tyr^326^ is at the C terminus of the conserved NP*XX*Y motif of GPCR seventh transmembrane domain, and its substitution to Ala leads to a dramatic sequestration defect and a partial G protein-coupling defect. Importantly, the β_2_AR(Y326A) variant is capable of full stimulation of adenylyl cyclase activity (28). We therefore investigated the agonist- and GRK2-dependent phosphorylation of the β_2_AR(Y326A) variant. Co-transfection of GRK2/Gβγ led to ∼10-fold stimulation of agonist-induced receptor phosphorylation at the Ser(P)^355^/Ser(P)^356^ phosphosites (Fig. 6*A*), consistent with previous results (27, 33, 39).

We next surveyed the ability of GRK2 mutants to phosphorylate this receptor. For most mutants, the ability of each GRK2 mutant to phosphorylate the β_2_AR(Y326A) in intact cells resembled its *in vitro* activity toward Rho (Fig. 6*B*). The L4A, V7E, D10R, Y13A, A16V, and M17A variants lost 75–95% of the WT cell-based phosphorylation activity (comparable with the kinase-deficient K220R variant) and were more impaired than in the *in vitro* Rho phosphorylation assay (Fig. 3*A*). It is possible that the β_2_AR(Y326A) mutant exaggerates the GRK2 defects. The M15A mutation again displayed no decrease in activity, mimicking its behavior in the Rho phosphorylation assay. The D3A variant, which showed a 2-fold defect in Rho phosphorylation, also did not exhibit a defect in β_2_AR phosphorylation. The V477D and I485A AST variants were as deficient as the D10R or Y13A variants in phosphorylation of the β_2_AR. These data suggest that variants L4A, V7E, D10R, Y13A, M15A, M17A, V477D, and I485 exhibited an overall consistency in their ability to phosphorylate Rho *in vitro* and the β_2_AR in intact cells. In contrast, D3A and A16V displayed subtle differences that may be attributed to receptor specificity, the assay milieu, or both.

##### Validation of a BRET-based α_2A_AR Recruitment Assay

**FIGURE 7.**
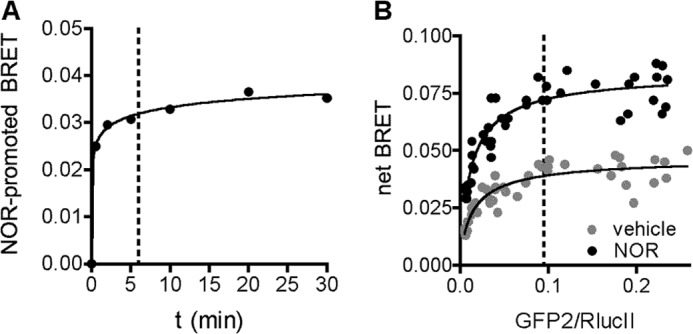
**Time course and titration of RlucII-GRK2 recruitment to NOR-activated α_2A_AR-GFP^2^ as measured by BRET.** *A*, time course. HEK293T cells transfected with α_2A_AR-GFP^2^ (1 μg) and WT RlucII-GRK2 (250 ng) were treated with 100 μm NOR at the indicated times. Data are expressed as NOR-promoted BRET and are the mean ± S.E. of three independent experiments carried out in triplicate. The *dashed line* represents the time (6 min) at which BRET data in Fig. 8 were collected. *B*, BRET titration. Cells transfected with RlucII-GRK2 and increasing amounts of α_2A_AR-GFP^2^ were incubated with or without NOR for 6 min. Data are expressed as net BRET values that correspond to BRET measurements that have been subtracted from the background BRET signal originating from luciferase alone. Values obtained from three independent experiments were pooled and plotted as individual data points. *Dashed lines* represent the time and acceptor/donor ratio, respectively, at which BRET experiments for Fig. 8 were performed.

One of the primary goals of this work was to distinguish residues on GRK2 that are important for direct interaction with GPCRs. This interaction is difficult to measure *in vitro* because GRK2 is expected to bind weakly to GPCRs and binds strongly to negatively charged phospholipids, which are required along with Gβγ for GRK2 activity (40). Thus, we turned to a cell-based BRET assay to examine recruitment of GRK2 to an activated GPCR. Although we were able to measure phosphorylation of the β_2_AR(Y326A) by GRK2 in intact cells (Fig. 6*B*), we have thus far been unable to detect a significant ISO-stimulated BRET signal between β_2_AR-GFP, β_2_AR-Venus, β_2_AR-GFP10, and luciferase-GRK2 or between β_2_AR-luciferase and GRK2-GFP10. The β_2_AR(Y326A) variant also did not yield a measurable ISO-promoted BRET response. However, we were able to detect agonist-dependent recruitment of GRK2 to the α_2A_AR, as indicated by the NOR-induced BRET signal observed between α_2A_AR-GFP^2^ and luciferase-GRK2 in a time course experiment (Fig. 7*A*). Co-expression of a constant level of RlucII-GRK2 with increasing concentrations of α_2A_AR-GFP^2^ led to an increase in basal and agonist-induced BRET signals in a saturable fashion (Fig. 7*B*), indicating a specific interaction between the two proteins. For all subsequent BRET experiments, acceptor/donor ratios yielding maximal BRET were used, and the readings were collected 6 min following receptor activation. These conditions provided stable signals and a good dynamic range.

**FIGURE 8.**
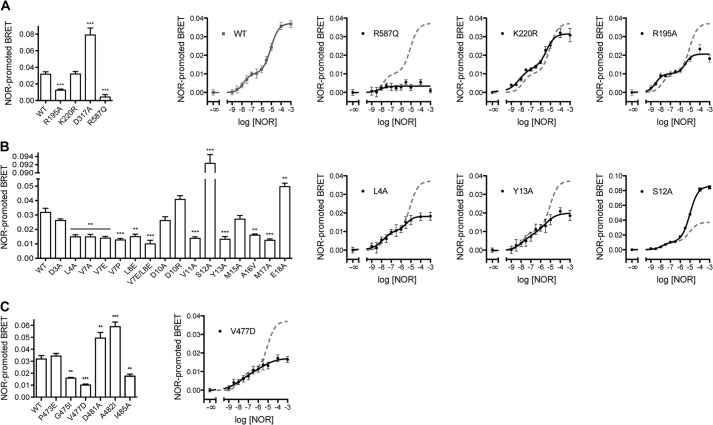
**Recruitment of RlucII-GRK2 variants to NOR-activated α_2A_AR-GFP^2^ by BRET.** *A*, GRK2 BRET controls; *B*, αN mutants; *C*, AST mutants. For *left-hand bar graph panels*, HEK293T cells were transfected as in Fig. 7*A* with α_2A_AR-GFP^2^ and WT or mutant RlucII-GRK2 with or without 100 μm NOR for 6 min. Data are expressed as agonist-promoted BRET and are the mean ± S.E. (*error bars*) of 3–5 independent experiments carried out in duplicate. One-way ANOVA with a Tukey's post-test was used to assess statistical significance *versus* WT. **, *p* < 0.01; ***, *p* < 0.001. Other *panels* in each *row* represent dose-response curves for selected mutants. HEK293T cells transfected with α_2A_AR-GFP^2^ and WT or mutant RlucII-GRK2 were treated in the presence of vehicle (−∞) or NOR at the indicated concentrations. For comparison purposes, the curve obtained with WT RlucII-GRK2 (shown as a *dashed line*) is superimposed on the data derived from the mutant. Data presented are means of NOR-promoted BRET ± S.E. of three independent experiments carried out in duplicate and are fitted to biphasic dose-response curves.

We wished to test whether the BRET-based GRK2 recruitment assay is dependent on the presence of liberated Gβγ subunits from the activated receptor, as would be expected from prior studies, and thus we assayed the R587Q mutant of GRK2, which is deficient in Gβγ binding but not in phosphorylation of soluble substrates (17). The NOR-promoted BRET signal of GRK2-R587Q was reduced by 90% relative to WT (Fig. 8*A*). As a positive control, we anticipated that catalytically inert mutants of GRK2 (such as K220R and D317A) would retain the agonist-induced BRET signal. The K220R and D317A variants are indeed efficiently recruited, with the D317A mutation being recruited 2-fold better than WT (Fig. 8*A*). The molecular basis for the increased recruitment of D317A is not clear, but the behavior of the K220R and D317A variants in the recruitment assay is consistent with the reported ability of catalytically inactive GRKs to serve as dominant negative mutants (29). Importantly, these data validate the use of the α_2A_AR-GFP^2^:RlucII-GRK2 pair as a BRET biosensor to identify specific mutations on GRK2 that impair its recruitment to an activated GPCR.

We next looked at the GRK2-R195A mutant. Arg^195^ is a residue on the small lobe of the kinase domain that, by analogy to Arg^190^ in GRK6, packs at the interface of the N-terminal helix and AST region and is dramatically disabled in its ability to phosphorylate Rho *in vitro* (900-fold *k*_cat_/*K_m_* defect) and β_2_AR in intact cells (21). We predict that the R195A mutation prevents small lobe, N-terminal helix, and AST interaction. The R195A mutation diminished the agonist-promoted BRET signal by 60% (Fig. 8*A*).

To test whether the residual BRET signal of the R195A mutant was due to Gβγ interaction, which would recruit the mutant to the vicinity of activated cell surface receptors, we measured the BRET signal of WT, R587Q, K220R, and R195A as a function of increasing concentrations of NOR. The WT and K220R variants exhibited a biphasic profile and could be fit by a two-site nonlinear regression analysis that highlighted the presence of a high-affinity component (EC_50,H_ = 15 nm) and a low-affinity component (EC_50,L_ = 7.9 μm) (Fig. 8*A*). For WT GRK2, the high- and low-affinity interactions account for ΔBRET values of ∼0.010 (27% of total BRET) and ∼0.027 (73% of total BRET), respectively. The impairment caused by the R587Q mutation, which prevents the binding of GRK2 to Gβγ, resulted in a nearly complete loss of the BRET signal. On the other hand, the dose-response curve for the R195A mutant exhibited a biphasic curve that retained the high-affinity interaction but a ∼60% reduced low-affinity interaction compared with WT. These results suggest that low agonist concentration promotes the GRK2/Gβγ interaction that accounts for ∼27% of the BRET signal, and higher agonist concentration is necessary to induce GRK2/receptor interaction that generates ∼73% of the agonist-induced BRET signal. Notably, if ∼27 and ∼44% (60% of the signal induced by high agonist concentration) account for the Gβγ and small lobe/N-terminal/AST-dependent BRET, respectively, ∼30% of the total agonist-induced BRET remains unaccounted for and might be attributed to interactions of the RH domain, the phosphoacceptor peptide, the N terminus, or other regions of GRK2 to the BRET signal. Collectively, these data validate the use of the α_2A_AR-GFP^2^:RlucII-GRK2 pair as a BRET biosensor to identify specific mutations in GRK2 that impair its recruitment to an activated receptor.

##### α_2A_AR Recruitment of GRK2 N-terminal and AST Variants

We then tested the ability of GRK2 variants with mutations in their N terminus for their ability to be recruited in the BRET assay with 100 μm NOR (Fig. 8*B*). In addition to mutants characterized in the *in vitro* and intact cell assays, we prepared the helix-breaking V7P variant to test the role of the α-helical nature of the N terminus and, because the L8A mutant enhanced Rho phosphorylation (Fig. 2), the L8E and the V7E/L8E variants to test whether elimination of hydrophobicity would fully disrupt receptor binding. The V7P and V7E/L8E variants reduced receptor recruitment 60 and 69%, respectively, to about the same extent as the R195A variant. Seven other mutants (L4A, V7A, V7E, L8E, V11A, Y13A, A16V, and M17A) exhibited statistically significant defects decreasing the agonist-promoted BRET by ∼55%. Investigation of the agonist dependence of the L4A and Y13A BRET revealed that these mutants exhibited a ∼70% decrease in the low-affinity BRET signal (Fig. 8*B*). Interestingly, the E18A mutant showed a strong potentiation of the recruitment to the activated receptor in line with what was observed for the phosphorylation of Rho in cell lysates (Fig. 2). Furthermore, although the S12A mutant was impaired in Rho phosphorylation, it exhibited a 2-fold potentiation of the total agonist-induced BRET and 2.6-fold stimulation of the low-affinity BRET signal (Fig. 8*B*). The D3A, D10A, D10R, and M15A variants did not show any significant difference in BRET signal compared with WT. The behavior of N-terminal mutants in the recruitment assay is therefore generally consistent with their activity in β_2_AR phosphorylation in cells (Fig. 5) with the exception of D10R, which was defective in β_2_AR phosphorylation. These results suggest that some GRK2 residues, such as Asp^3^ and Ser^12^, could make distinct interactions of variable importance with Rho, β_2_AR, and α_2A_AR.

The effects of AST mutations on receptor recruitment were then investigated (Fig. 8*C*). The P473E substitution had no impact on recruitment to activated α_2A_AR, whereas G475I and I485A showed 44–50% reduction of the BRET signal. The V477D mutation was the most deleterious of all tested, and its high-agonist concentration BRET signal was inhibited 74% (Fig. 8*C*). These results paralleled the reduced ability of these AST variants to phosphorylate Rho *in vitro* (Fig. 4) and β_2_AR in intact cells (Fig. 6). Mutants D481A and A482I potentiated GRK2 recruitment to the activated receptor 53–84%. This is in contrast with the slightly reduced ability of A482I (Fig. 3*A*) or the insignificant decrease in the ability of D481A to phosphorylate Rho *in vitro* (Table 2) (22).

**TABLE 2:**
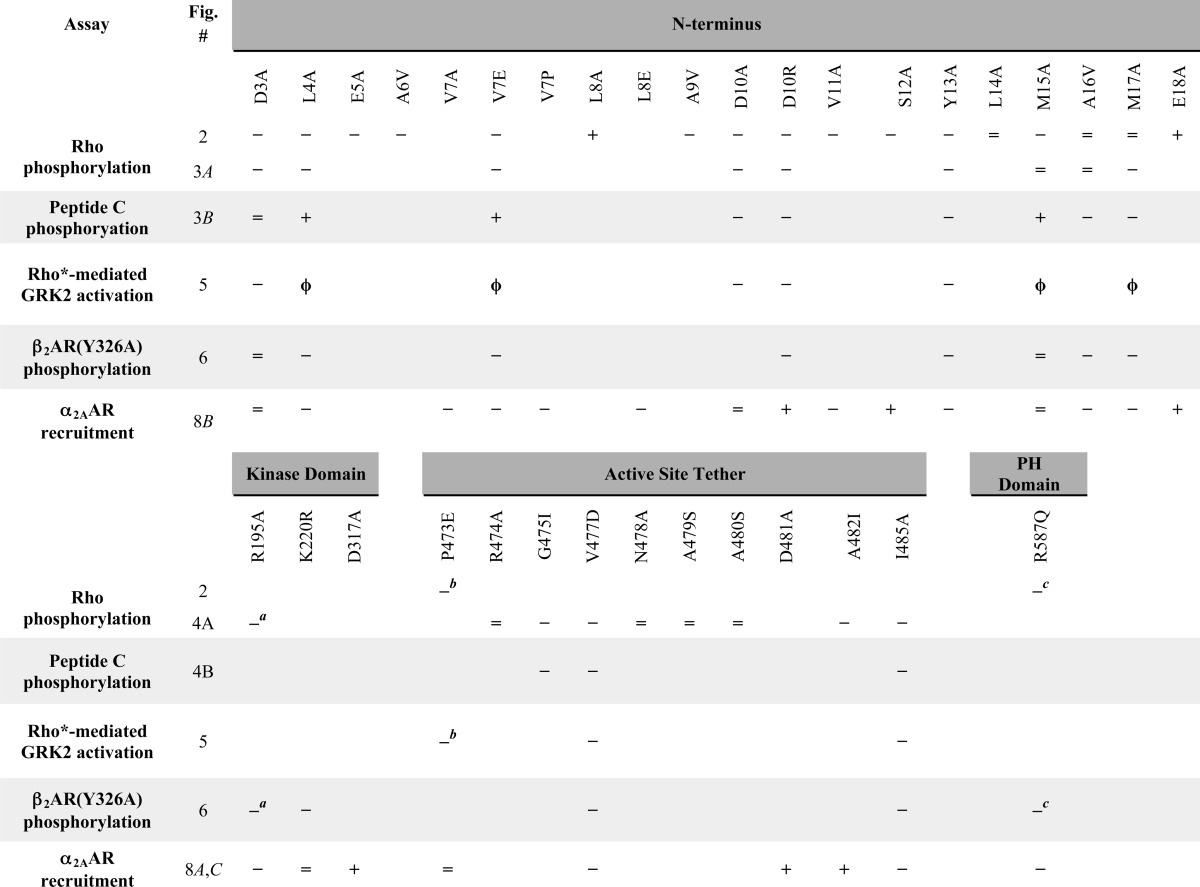
**Comparison of GRK2 mutants in various assays** −, =, +, or φ, effect on response: impairment, neutrality, potentiation, or weak impairment, respectively.

^*a*^ Ref. 21.

^*b*^ Ref. 22.

^*c*^ Ref. 17.

## DISCUSSION

The two major goals of this work were 1) to assess the roles of specific GRK2 αN and AST residues in forming a potential receptor-docking site and 2) to determine how individual residues contribute to receptor interaction, allosteric activation, or both. To this end, we created a homology model of activated GRK2 based on the GRK6-sangivamycin structure (Fig. 1). In this model, αN nestles against the small lobe and makes several AST contacts. Likewise, the AST makes both small lobe and αN contacts. Although any solvent-exposed residue in this model could participate in direct receptor interactions, we only found one locale of GRK2 mutants whose sole defect seems to be in receptor interaction, as opposed to allosteric activation. αN mutants L4A and V7E, whose side chains project away from the AST or small lobe, were defective in Rho phosphorylation but not peptide C phosphorylation (Table 2). The L4A mutant displayed *K_m_* but not *k*_cat_ defects in Michaelis-Menten kinetic studies with Rho as substrate (Table 1), again consistent with a role in receptor docking. Based on the homology model, mutation of these αN residues is not likely to impact the helical structure of the N terminus, the GRK2 tertiary structure, or the overall structure of the proposed docking site. Based on these considerations, we propose that the surface composed of Leu^4^, Val^7^, Leu^8^, Val^11^, and Ser^12^ constitutes a GPCR docking site.

In contrast, some residues seem to play a role in receptor-mediated kinase activation because mutation of these positions exhibited profound defects on both receptor and peptide C phosphorylation (Figs. 3 (*A* and *B*) and 4 (*A* and *B)* and Table 2). Mutants with these characteristics, D10R, Y13A, G475I, V477D, and I485A, map to both αN and AST. M17A was not defective in Rho phosphorylation but was impaired in β_2_AR(Y326A) and peptide C phosphorylation and thus might also fall in this category (Table 2). Our GRK6-based homology model of GRK2 predicts that Tyr^13^ contacts Val^477^ of the AST as well as the kinase small lobe, and Gly^475^ packs against Tyr^13^, Leu^14^, and Met^17^ of αN (Fig. 1, *B* and *C*). Ile^485^ is predicted to pack against the small lobe but does not directly interact with the αN helix. In general, GRK2 αN and AST variants behave similarly to their GRK1/6 cognates (12, 21) (Table 3), and it is therefore likely that interaction of the αN and AST regions of GRK2 is necessary to form the docking site and the closed kinase.

**TABLE 3:**
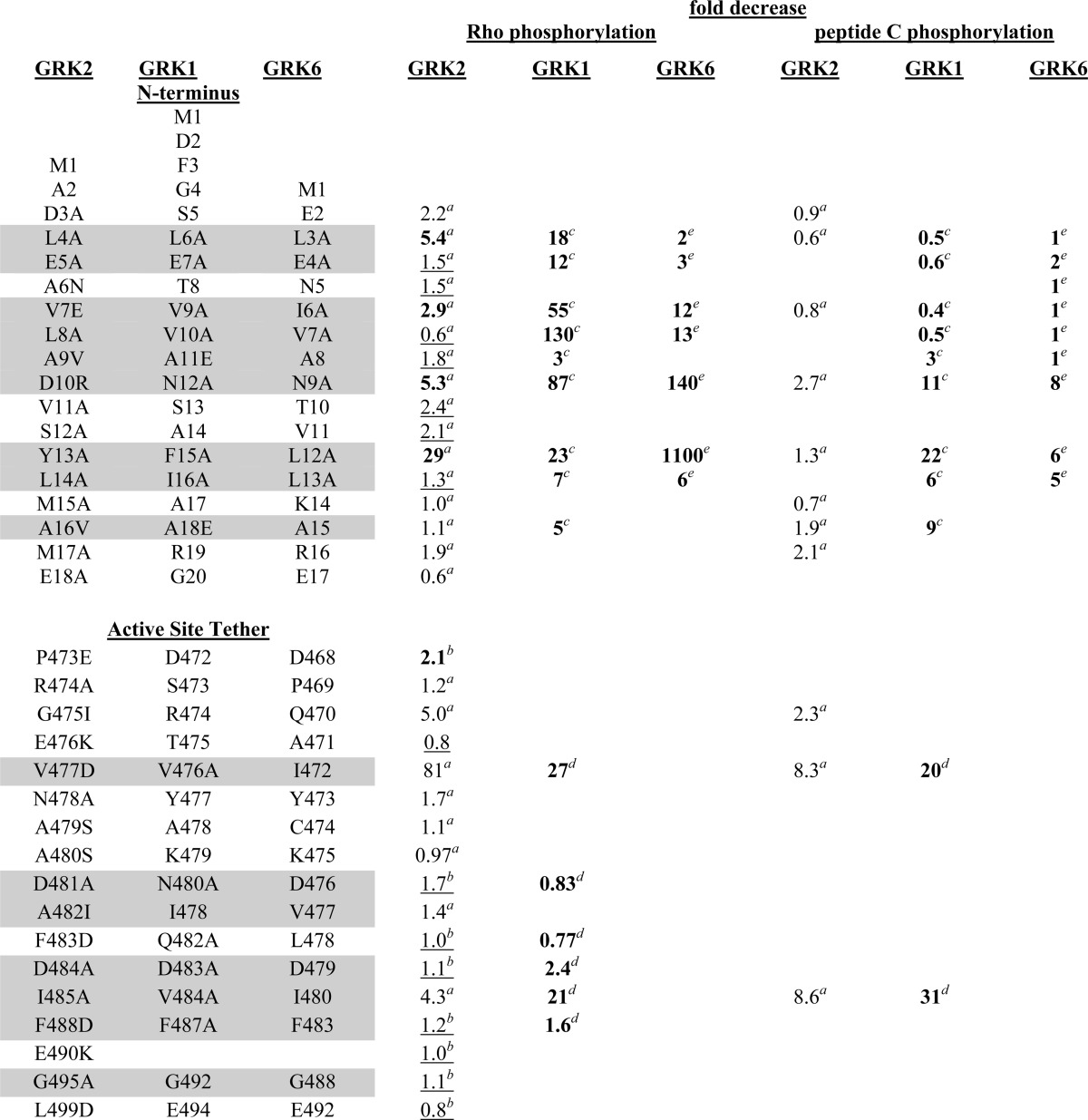
**Comparison of Rho and peptide C phosphorylation by GRK1, GRK2, and GRK6 mutants** Values derived from Michaelis-Menten kinetic data are in boldface type, values from purified GRK assayed at phosphoacceptor *K_m_* are in regular type, and values values from GRK in COS-7 or High 5 lysate assayed at phosphoacceptor *K_m_* are underlined. Gray shading indicates homologous residues conserved across three GRK subfamilies.

^*a*^ This work.

^*b*^ Ref. 22.

^*c*^ Ref. 13.

^*d*^ Ref. 21.

^*e*^ Ref. 12.

The use of assays that measure cell-based GRK2 activity was key to extending these studies beyond what has been shown by analogous studies of GRK1 and GRK6. The first of these assays (33) was a modification and optimization of published assays (21, 38) that allowed the quantitative assessment of the ability of exogenous GRK2 variants to phosphorylate transfected β_2_AR(Y326A) in COS-7 cells. The results largely replicated the defects for the variants that were observed *in vitro*. For some variants (V7E, D10R, Y13A, A16V, M17A, I485A), the defect in phosphorylating β_2_AR(Y326A) in intact cells was more pronounced than the defect in phosphorylating Rho *in vitro* (Figs. 3*A* and 6*B*). It is possible that these positions play a more important role in β_2_AR than Rho phosphorylation or, alternatively, that the β_2_AR(Y326A) variant exaggerates the GRK2 defects.

The second assay developed for this study examined the NOR dependence of GRK2 recruitment to α_2A_AR as measured by BRET and proved to be a surprisingly rich source of novel information. The BRET signal in response to increasing concentrations of NOR was biphasic, displaying both high-affinity (EC_50_ of 15 nm) and low-affinity phases (EC_50_ ∼8 μm) (Fig. 8) that we propose reflect the interaction of GRK2 with Gβγ (and probably phospholipids) and with receptor, respectively. The R587Q mutant, which is defective in Gβγ-dependent stimulation of Rho phosphorylation *in vitro* and GRK2-dependent phosphorylation of β_2_AR(Y326A) and μ-opioid receptor in intact cells (17), had a nearly abolished BRET response, whereas the kinase small lobe mutant R195A and other variants severely impaired in receptor phosphorylation *in vitro* retained a biphasic profile with a much reduced high-agonist concentration component. Thus, we hypothesize that this BRET assay can detect both interactions with Gβγ (low-NOR concentration phase) and with receptor (high-NOR concentration phase). The ∼30% of the high-agonist BRET signal that persists when the small lobe/αN/AST interactions are disrupted (*i.e.* in R195A mutants) could reflect the fact that other regions of GRK2 still retain the ability to bind to receptor, such as the kinase large lobe binding to the third intracellular loop of α_2A_AR, which is the site of phosphorylation, the RH domain, or some other region of GRK2 binding to the receptor. It could also represent residual binding of the N-terminal region to the receptor despite the fact that these mutants fail to undergo allosteric activation. It will be interesting to see how ligands that are biased toward G protein coupling *versus* β-arrestin signaling (41, 42) affect the detection of the low-affinity component of GRK2 recruitment to activated receptors.

The high-agonist concentration BRET signal in the GRK2 recruitment assay was diminished by various αN and AST mutants and potentiated by the αN S12A mutation (Fig. 8*B*). The high NOR requirement to detect this putative GRK2/receptor interaction is consistent with the observation that homologous desensitization requires high receptor occupancy by agonist (43). Moreover, phosphorylation of the β_2_AR at short time periods by endogenous kinases in HEK293 cells requires high agonist concentration (38, 44). The simplest explanation is that GRK2 αN/AST interaction provides a docking site on receptors that is captured by and comprises a major component of the high-NOR concentration BRET signal that we observe.

Recruitment of GRK2 mutants to α_2A_AR in intact cells paralleled their ability to phosphorylate receptors *in vitro* and in intact cells with the exception of the D10R/A variants, which were defective in *in vitro* Rho and peptide C phosphorylation, receptor-promoted phosphorylation of peptide C, and intact cell β_2_AR phosphorylation but were normal in recruitment to α_2A_AR (Table 2). In our GRK6-based homology model (Fig. 1*B*), the Asp^10^ side chain makes van der Waals interactions with Val^477^ and ionic interactions with Arg^195^. To align these observations, we predict that D10R or D10A variants would retain hydrophobic contacts with the AST residue Val^477^, but their interactions (or lack thereof) with the critical small lobe residue Arg^195^ would hinder kinase domain activation. If true, then receptor docking can be uncoupled from kinase domain closure. However, we cannot rule out the possibility that Asp^10^ plays a more important role in forming interactions with the β_2_AR and Rho than with the α_2A_AR.

Our data are partially consistent with the study of Pao *et al.* (11), which reported that the GRK2-D3K, L4A, and D10A mutants were severely impaired in β_2_AR phosphorylation but not tubulin phosphorylation. Whereas we observed partial activation defects with L4A and complete defect with D10A (Figs. 3*B* and 5), these authors also showed that GRK2-D3K, -L4A, and -D10A were completely defective in Rho*-stimulated phosphorylation of the RRRASAAASAA peptide. Their study concluded that the N terminus of GRK2 is involved in bridging interactions between phospholipids and the kinase domain in a GPCR-dependent but not agonist-dependent manner. Our cell-based assays cannot distinguish between this possibility and the direct receptor interaction we propose for this same region. The fact that some mutants display receptor-specific effects (see below) argues against these residues playing a role in phospholipid interactions. However, resolution of this question will probably require structure determination of a GRK-GPCR complex.

Vertebrate GRKs are divided into three subfamilies based on homology and gene structure (45, 46). Each subfamily has highly conserved N-terminal and AST regions that, although homologous, exhibit distinct sequence differences. Thus, it can be anticipated that they will form similar tertiary structures but that distinct residues may be responsible for the GPCR selectivity reported for some GRKs. For example, the β_2_AR is a far more potent activator of GRK2 than is Rho (47) and although GRK2, -3, -5, and -6 are each present in pituitary GH3 cells, GRK2 seems to be the GRK responsible for phosphorylating the thyrotropin-releasing hormone receptor (48). *In vitro* GRK1 phosphorylates Rho more efficiently than does GRK2 or GRK5 (49), whereas GRK2, GRK5, and GRK6 phosphorylate other GPCRs (β_2_AR and m2 muscarinic cholinergic receptors) more efficiently than they phosphorylate Rho (50–52). Although our Rho phosphorylation results with GRK2 are generally similar to those obtained for GRK1 and GRK6, there were some notable differences (Table 3). For example, the GRK2-Leu^4^ cognate is more important in GRK1 and GRK2 than it is in GRK6, the GRK2-Glu^5^ cognate is more important in GRK1 than it is in GRK2 and GRK6, and the GRK2-Tyr^13^ cognate is even more important in GRK6 than it is in GRK1 or GRK2. The L8A mutant of GRK2 actually enhances receptor phosphorylation, whereas the corresponding V10A and V7A cognate mutants of GRK1 and GRK6, respectively, are severely impaired in this assay. These observations suggest that these GRK isoforms make distinct GPCR interactions.

With the caveat that three GPCRs were tested in three types of assays in this study, some GRK2 mutants behaved consistently across assays/GPCRs, whereas others showed distinct profiles. Two of these mutants, V477D and I485A, have been assayed by *in vitro* phosphorylation of Rho, by *in vitro* phosphorylation of β_2_AR (22), and in intact cell β_2_AR phosphorylation. The behaviors of these two mutants are consistent across these three assays. Therefore, we predict that the differential effects of various GRK2 mutants in phosphorylation assays (Table 3) may reflect receptor-specific interaction. D3A showed a 50% defect in Rho phosphorylation and Rho-dependent peptide C phosphorylation but no defect in β_2_AR phosphorylation. Conversely, A16V and M17A showed much greater defects in β_2_AR than Rho phosphorylation (Figs. 3, 5, and 6). Ideally we would compare GRK binding to and phosphorylation of various GPCRs in the same battery of intact cell and *in vitro* assays. Only with such assays can we rigorously assess receptor specificity.

Some of our variants exhibited gain of function, which gives additional insight into which positions are involved in direct interactions with receptors and, potentially, in mandating receptor selectivity. The V8A and E18A mutants exhibited elevated Rho phosphorylation (Fig. 2), and the E18A, S12A, D481A, and A482I variants potentiated NOR-induced recruitment to α_2A_AR (Fig. 8, *B* and *C*). Each of these residues is predicted to be fully or partially solvent-exposed (Fig. 1, *B* and *C*), and each of the substitutions decreases negative charge and/or increases hydrophobicity at these positions. These results suggest that Ser^12^, Glu^18^, Asp^481^, and Ala^482^ play a role in direct interactions with receptors. Alternatively, because two of these residues bear negative charge, the mutations may also reduce repulsion with the negatively charged phospholipid bilayer. Regardless, these results demonstrate that it is possible to create GRK2 variants with enhanced function. The variable effects of mutants (*e.g.* D481A and A482I) on Rho phosphorylation *versus* recruitment to α_2A_AR suggest that it is also be possible to create GRK variants with altered GPCR specificity.

In summary, our work implies that residues throughout the length of the GRK2 αN helix play a role in receptor interaction and/or substrate phosphorylation. Our results are consistent with the idea that the αN/AST interaction, mediated directly or indirectly by GRK2 Tyr^13^, Met^17^, Gly^475^, Val^477^, and Ile^485^ (Fig. 1, *B* and *C*), is necessary for kinase domain activation and that this interaction is required for efficient docking to an activated receptor. Our work suggests that GRK2 Leu^4^, Val^7^, Leu^8^, Val^11^, and Ser^12^ directly interact with GPCRs. Finally, our work suggests that Asp^10^ may be more critical for kinase domain activation than for forming the GPCR docking site. In comparison with previous studies on GRK1 and GRK6, our results with GRK2 highlight differences between GRK subfamilies that may dictate receptor preference. Although BRET- and FRET-based GRK/GPCR recruitment assays have been reported (25, 53, 54), our GRK2 recruitment assay to the activated α_2A_AR seems to tease apart a low-agonist concentration membrane localization event from a high-agonist concentration receptor localization event. This experimental approach should prove very useful in further dissecting the mechanism of GRK2 activation and understanding the regulation of other GPCRs.

## Footnotes

4 The abbreviations used are:

GPCR: G protein-coupled receptor
GRK: GPCR kinase
AST: active site tether
BRET: bioluminescence resonance energy transfer
HEK293: human embryonic kidney 293
ISO: isoproterenol
NOR: norepinephrine
RH: RGS homology
Rho: rhodopsin
α_2A_AR: α_2A_-adrenergic receptor
β_2_AR: β_2_-adrenergic receptor
NCB: Ni^2+^-column buffer
HCB: High S column buffer
ANOVA: analysis of variance
PDB: Protein Data Bank.
